# Long-Term Survival of Very Elderly Patients with Heart Failure Managed in a Comprehensive Heart Failure Management Unit

**DOI:** 10.3390/geriatrics11050119

**Published:** 2026-09-03

**Authors:** Sonia González-Sosa, Alba Rodríguez-Quintana, Pablo Santana-Vega, Jose A. Rodríguez-González, José M. García-Vallejo, Jorge Arencibia-Borrego, Alicia Conde-Martel

**Affiliations:** 1 Internal Medicine, Hospital Universitario de Gran Canaria Doctor Negrín, 35010 Las Palmas de Gran Canaria, Spain; sgonsos@hotmail.com (S.G.-S.); jberto6@gmail.com (J.A.R.-G.); jogava90@gmail.com (J.M.G.-V.); jab1jab1@gmail.com (J.A.-B.); 2 Department of Clinical Sciences, Universidad de Las Palmas de Gran Canaria, 35016 Las Palmas de Gran Canaria, Spain; infbalbarodriguez@gmail.com (A.R.-Q.); pablosv2000@gmail.com (P.S.-V.)

**Keywords:** heart failure, elderly patients, survival, comorbidities, readmission, NT-proBNP

## Abstract

**Background**: Heart failure (HF) is the leading cause of hospitalisation in older adults, with incidence increasing markedly with age. However, long-term outcome data in patients aged ≥85 years remain limited. We evaluated survival and predictors of mortality in this very elderly HF population enrolled in a structured outpatient programme. **Methods**: A retrospective observational study was conducted, including patients aged ≥85 years assessed at a Comprehensive HF Management Unit between 2012–2023. Demographic characteristics, comorbidities, functional (Barthel) and cognitive (Pfeifer) status, laboratory parameters, hospitalisations, and survival status were collected. Long-term survival was estimated using the Kaplan–Meier method, and predictors of mortality were analysed using Cox regression models. **Results**: A total of 514 patients were included (mean age 88.4 ± 3 years, 46.1% male); 340 (66.1%) died during follow-up. Survival was 77% at 1 year, 50% at 2.5 years, and 25% at 5 years. Mortality was associated with diabetes (*p* = 0.038), advanced chronic kidney disease (*p* = 0.008), dementia (*p* < 0.001), neoplasia (*p* = 0.007), ischaemic heart disease (*p* < 0.001), history of HF (*p* = 0.043), NYHA III–IV (*p* < 0.001) and elevated NT-proBNP (*p* < 0.001). Worse functional and cognitive status, higher comorbidity according to the Charlson Comorbidity Index and readmission at one year (all *p* < 0.001) were also related to mortality. In the first multivariable model, worse cognitive and functional status, a higher Charlson comorbidity index, NYHA class III–IV, and high NT-proBNP were independent predictors of mortality. When 1-year HF readmission was added to the model, the Charlson index (HR:1.10; 95%CI 1.01–1.19), high NT-proBNP (HR:1.76; 95%CI 1.30–2.38) and HF readmissions within 1 year (HR:2.68; 95%CI 1.72–4.16) remained independently associated with mortality. **Conclusions**: In patients aged ≥85 years with HF, survival was poor, with only half alive at 2.5 years and 25% at 5 years. Comorbidity burden, high natriuretic peptides, and early readmission independently predicted mortality, underscoring the importance of comprehensive assessment in this population.

## 1. Introduction

Heart failure (HF) represents a major public health problem worldwide, associated with high morbidity, mortality, and healthcare costs [1]. It affects more than 64 million people globally and its prevalence is expected to increase due to population ageing and improved survival from cardiovascular disease [1]. HF prevalence increases markedly with age [2], and in Western countries it is one of the leading causes of hospitalisation among older adults [3].

HF predominantly affects older adults, whose clinical, pathophysiological, and epidemiological characteristics differ substantially from those of younger individuals [4]. Ageing promotes structural cardiac, vascular, and valvular changes—including myocardial fibrosis, diastolic dysfunction, and arterial stiffening—that predispose to HF [5,6]. In addition, clinical manifestations may often be atypical or subtle and may remain unrecognised until severe decompensation occurs [4].

Comorbidities are highly prevalent in patients with HF, and most individuals present with multiple coexisting conditions. Their presence is particularly relevant because they can hinder diagnosis by masking symptoms, interfere with treatment, contribute to progression, and therefore have a negative impact on prognosis [7]. Beyond comorbidity, older patients are commonly affected by polypharmacy, as well as functional and cognitive impairment, highlighting the need for a comprehensive assessment [8].

The limited clinical evidence available for the management of these complex patients [8] together with the high healthcare burden associated with HF [9], has led to the development of care models based on structured and continuous management such as the Comprehensive Management Units for Patients with Heart Failure (UMIPIC programme), designed for patients with multimorbid HF and advanced age [10,11]. Although previous studies have demonstrated reductions in hospital admissions and emergency department visits among patients followed in these programmes [11], the clinical course of very elderly patients (≥85 years) with HF remains poorly characterised, despite representing the fastest growing segment of the HF population. Moreover, available evidence in this age group has largely focused on short-term outcomes, frequently following hospitalisation for acute decompensation, whereas long-term survival and its determinants in patients managed within structured outpatient programmes have been far less explored [12,13,14]. A better understanding of their prognosis and clinical profile may help optimise management and improve care strategies.

Therefore, the aim of this study was to evaluate long-term survival of patients aged ≥85 years with HF included in a structured outpatient follow-up programme, as well as to describe their clinical characteristics and to identify independent prognostic factors.

## 2. Materials and Methods

### 2.1. Design and Study Population

We conducted a single-centre retrospective observational study that included all consecutive patients aged 85 years or older who were evaluated at the Comprehensive Management Unit for Patients with Heart Failure (UMIPIC) of a tertiary hospital from July 2012 to May 2023, except for those meeting the exclusion criteria described below. The sample size was 514 patients. Patients were referred to the unit mainly after discharge from an HF hospitalisation or from the emergency department following a decompensation not requiring admission, and less frequently from other hospital services or from primary care. There were no strict referral criteria; however, referral was based on the responsible physician’s judgement and typically involved patients with more advanced HF that was difficult to manage in primary care and/or with frequent decompensations or admissions, whose functional status still allowed transfer to the hospital. Patients were followed from the first visit until death or the last recorded contact; the median follow-up was 22.6 months (interquartile range [IQR] 10.6–40.3 months).

### 2.2. Inclusion and Exclusion Criteria

Patients were eligible if they had a diagnosis of HF according to the European Society of Cardiology heart failure guidelines [15], were aged ≥85 years at the time of the first visit, and had their first visit between July 2012 and May 2023. Patients were excluded if they did not meet the age criteria or were lost to follow-up after the first visit, defined as the absence of any subsequent clinical contact or recorded information after baseline. Of 1519 patients assessed at a first visit during the study period, 993 were excluded because they did not meet the age criterion and 12 because they had no contact after the first visit.

### 2.3. Study Variables

Sociodemographic characteristics were collected, including sex, age, place of residence and cohabitants. The comorbidities included in the Charlson Comorbidity Index [16] and other conditions not included in the index, such as arterial hypertension, dyslipidaemia, and atrial fibrillation, as well as lifestyle factors (e.g., smoking, alcohol use), were also recorded at the baseline visit. The non-age-adjusted version of the Charlson Comorbidity Index was used. Functional status was assessed using the Barthel Index [17] and cognitive status using the Pfeiffer Short Portable Mental Status Questionnaire [18]. Both scales were administered at study entry by trained staff as part of the unit’s standardised assessment protocol. No formal frailty assessment instrument was used.

With regard to heart failure, left ventricular ejection fraction (LVEF) measured by echocardiography and the presence of valvular heart disease were recorded. The functional class according to the New York Heart Association (NYHA) was also assessed [19]. HF was classified as new-onset when it was first diagnosed at the time of referral to the unit in patients with no prior history of HF—whether or not the presentation involved an episode of decompensation—as opposed to chronic HF, defined by a previously established diagnosis. HF was categorised according to LVEF as reduced (HFrEF, LVEF ≤ 40%), mildly reduced (HFmrEF, 41–49%) or preserved (HFpEF, LVEF ≥ 50%) [15]. LVEF was obtained from the transthoracic echocardiogram closest to the baseline visit, applying the same approach to all patients. Whenever available, LVEF was quantified using the biplane Simpson’s method; when this was not available, the Teichholz method was used; and when no quantitative measurement was feasible, LVEF was estimated visually.

Laboratory data were collected at the baseline visit (start of follow-up), including complete blood count, renal function, and natriuretic peptides (NT-proBNP). Pharmacological treatment at 3 months after the initial visit was also recorded.

Hospital admissions and emergency department visits due to HF during the year before and the year following the first visit were also recorded. Finally, the date of last follow-up and vital status (alive or deceased) were documented.

### 2.4. Statistical Analysis

Statistical analysis was performed with the SPSS software (Statistical Package for the Social Sciences, IBM Corp, Version 29.0, Armonk, NY, USA). Categorical variables were expressed as frequencies and percentages, and continuous variables as mean and standard deviation (SD) or as median and interquartile range [IQR] depending on whether the variables followed a normal distribution, assessed using the Kolmogorov–Smirnov test.

The optimal NT-proBNP cut-off point for predicting mortality was calculated according to the Youden index, which is the value that maximises the sum of sensitivity and specificity [20], yielding a threshold of 2285 pg/mL. NT-proBNP was entered as a log10-transformed variable in the univariable analyses, and as a dichotomised variable at the Youden-derived cut-off (>2285 vs. ≤2285 pg/mL) in the multivariable Cox models, to calculate Hazard Ratios (HR) and their confidence intervals.

To assess the relationship between different variables and mortality, univariate analyses were performed. Categorical variables were compared using the Chi-square or Fisher’s exact test, and continuous variables using Student's *t*-test or Mann–Whitney U test, depending on their distribution. Survival was estimated using the Kaplan–Meier method. The Log-rank test was used to identify the variables associated with survival. The variables that showed a significant association in the univariate analysis (*p* < 0.05) were included in a multivariable Cox proportional hazards regression model using a forced-entry method. Individual comorbidities were not included, as overall comorbidity burden was represented by the Charlson Comorbidity Index; infiltrative aetiology was excluded owing to its small sample size; and age and sex were included as clinically relevant adjustment variables regardless of their univariable significance. The proportional hazards assumption was assessed by testing time-dependent covariates (interaction of each covariate with the logarithm of survival time); the main covariates showed time-dependent effects that attenuated during follow-up, so the reported hazard ratios represent average effects over the follow-up period. Missing data were handled by complete-case (listwise) analysis: patients with a missing value in any variable included in a given multivariable model were excluded from that model, and no imputation was performed. The baseline model included 425 patients (277 deaths), and the model additionally including 1-year HF readmission included 318 patients (196 deaths). The number of events per variable exceeded the recommended minimum of ten in both multivariable models (approximately 31 and 20 events per variable in the baseline and readmission models, respectively), calculated from the deaths available after complete-case selection. A value of *p* < 0.05 was considered statistically significant.

### 2.5. Ethical Aspects

This study was approved by the Ethics and Clinical Research Committee of the Hospital Universitario de Gran Canaria Dr. Negrín (Ethics Committee approval number: 2023-507-1). The information was anonymised in the database to ensure confidentiality.

## 3. Results

### 3.1. Baseline Characteristics

A total of 514 patients were included, with a mean age of 88.4 years (SD 2.9), median of 88 (interquartile range [IQR] 86–90), and a range of 85 to 101 years. Of these, 237 (46.1%) were male and 277 (53.9%) were female. Eighty-five patients (16.5%) lived alone and 126 (24.5%) lived with their spouse (Table 1).

Multimorbidity was marked, with a median Charlson comorbidity index of 3; hypertension was almost universal (95.9%); and more than half of the patients had chronic kidney disease, atrial fibrillation and anaemia (Table 1). 

Cognitive impairment (Pfeiffer test ≥ 3 errors) was present in 104 patients (20.2%) and functional impairment (Barthel < 60 points) in 87 patients (16.9%) (Table 2).

### 3.2. Pharmacological Treatment and Heart Failure Characteristics

The most prescribed treatments (Table 3) were loop diuretics (89.7%), followed by beta-blockers (61.1%), oral anticoagulants (59.9%) and statins (52.9%). SGLT2 inhibitors were prescribed in 114 patients (22.6%) and ARNI in 52 (10.3%). By heart failure phenotype, ARNI and SGLT2 inhibitors were used more frequently in patients with reduced and mildly reduced ejection fraction than in preserved ejection fraction (ARNI: 40.0%, 24.5% and 2.6%; SGLT2 inhibitors: 38.6%, 32.1% and 18.4%, respectively), whereas beta-blockers (61.4%, 67.9% and 60.8%) and mineralocorticoid receptor antagonists (41.4%, 47.2% and 41.3%) were used similarly across phenotypes.

Regarding HF characteristics, HFpEF was the predominant phenotype (73.9%), followed by HFrEF (13.6%) and HFmrEF (10.3%); LVEF was unavailable in 2.1% of patients. The most frequent aetiology was hypertension (76.5%) and the most common valvular disease was mitral regurgitation in 53.1% (Table 4).

At baseline, 62.8% of patients (323) had a history of previous hospitalisation and 52.3% (269) had been hospitalised due to HF during the year prior to inclusion. During follow-up, 11.4% of patients (*n* = 43) were readmitted for HF within the first year. Overall, 340 patients (66.1%) died during follow-up (Table 4).

### 3.3. Long-Term Survival

**Figure 1:**
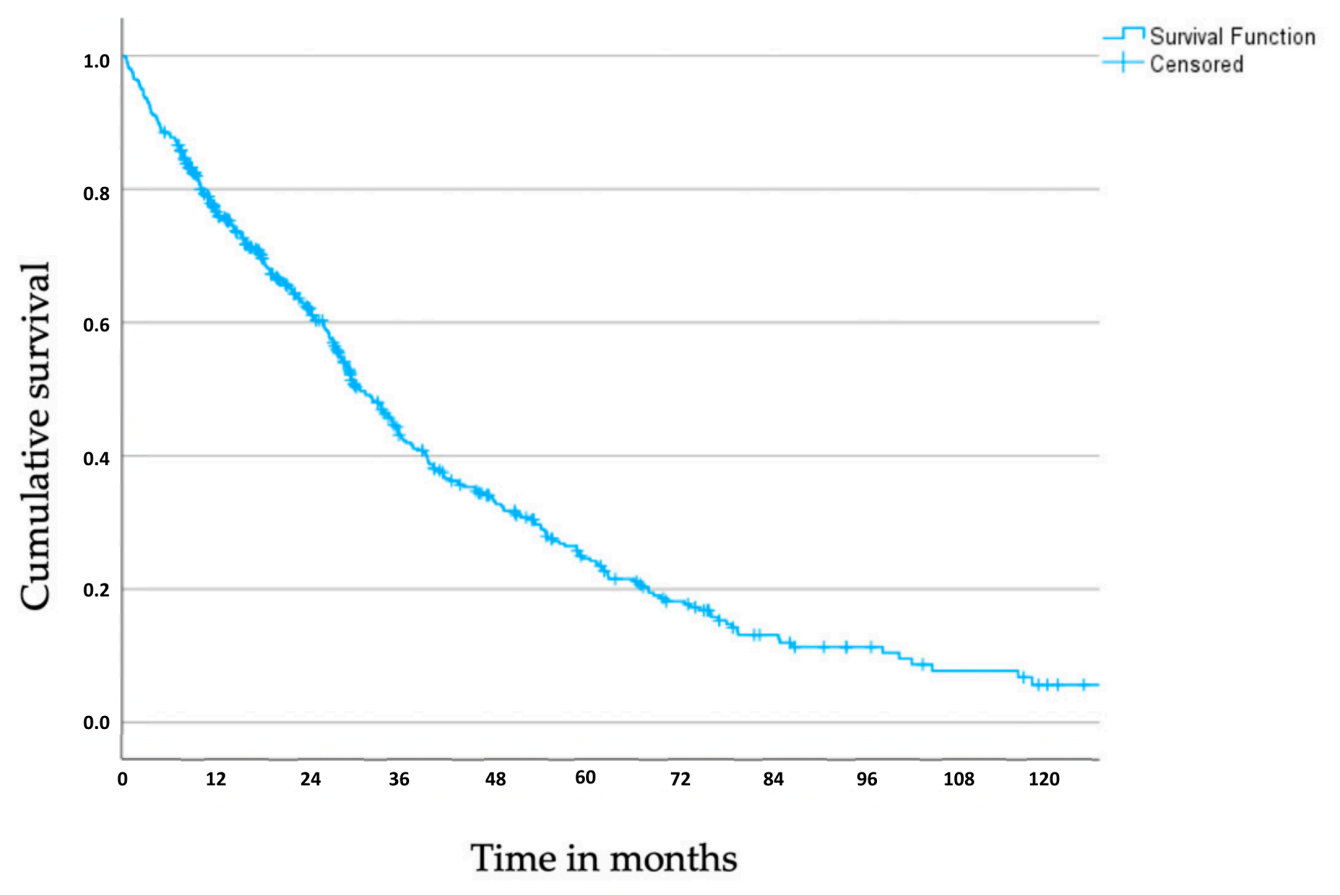
Kaplan–Meier survival curve of the study population during follow-up.

The median follow-up was 22.6 months (IQR: 10.6 to 40.3), with a mean follow-up of 29.5 months. The maximum follow-up time was 127.4 months. The median survival was 30.5 months and the mean was 41.3 months. Survival rates were 77% at 1 year, 50% at 2.5 years, and 25% at 5 years. Figure 1 shows the Kaplan–Meier survival curve.

### 3.4. Univariable Analysis of Factors Associated with Mortality

**Figure 2:**
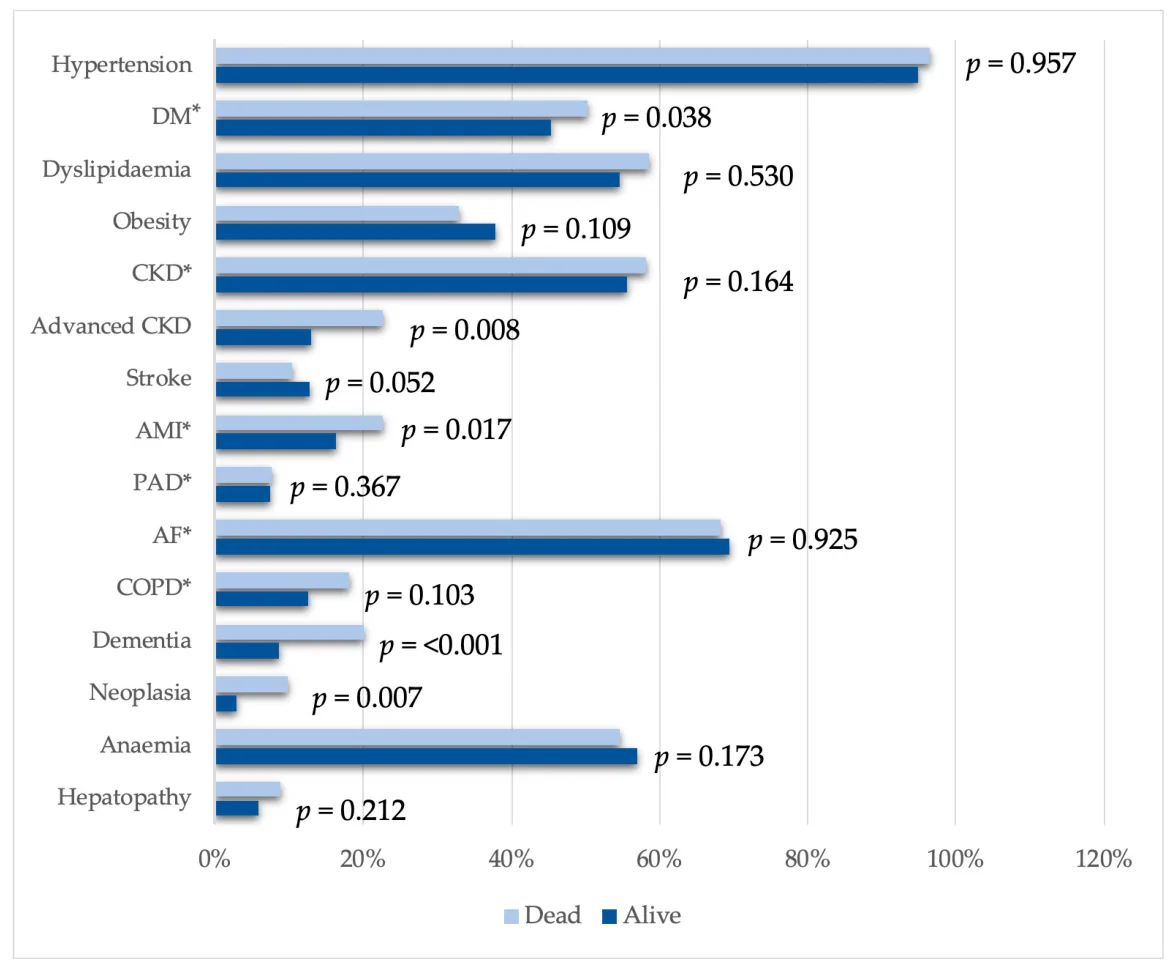
Prevalence of comorbidities according to survival status (in survivors and deceased patients). * DM: diabetes mellitus; CKD: chronic kidney disease; AMI: acute myocardial infarction; PAD: peripheral arterial disease; AF: atrial fibrillation; COPD: chronic obstructive pulmonary disease.

Comorbidities significantly associated with shorter survival were diabetes mellitus (HR: 1.26; 95% CI 1.01–1.56), advanced chronic kidney disease (eGFR < 30 mL/min/1.73 m^2^) (HR: 1.99; 95% CI 1.19–3.35), myocardial infarction (HR: 1.36; 95% CI 1.06–1.76), dementia (HR: 1.78; 95% CI 1.36–2.31) and neoplasia (HR: 1.65; 95% CI 1.15–2.36) (Table 1). Figure 2 shows the prevalence of different comorbidities among surviving and deceased patients.

**Figure 3:**
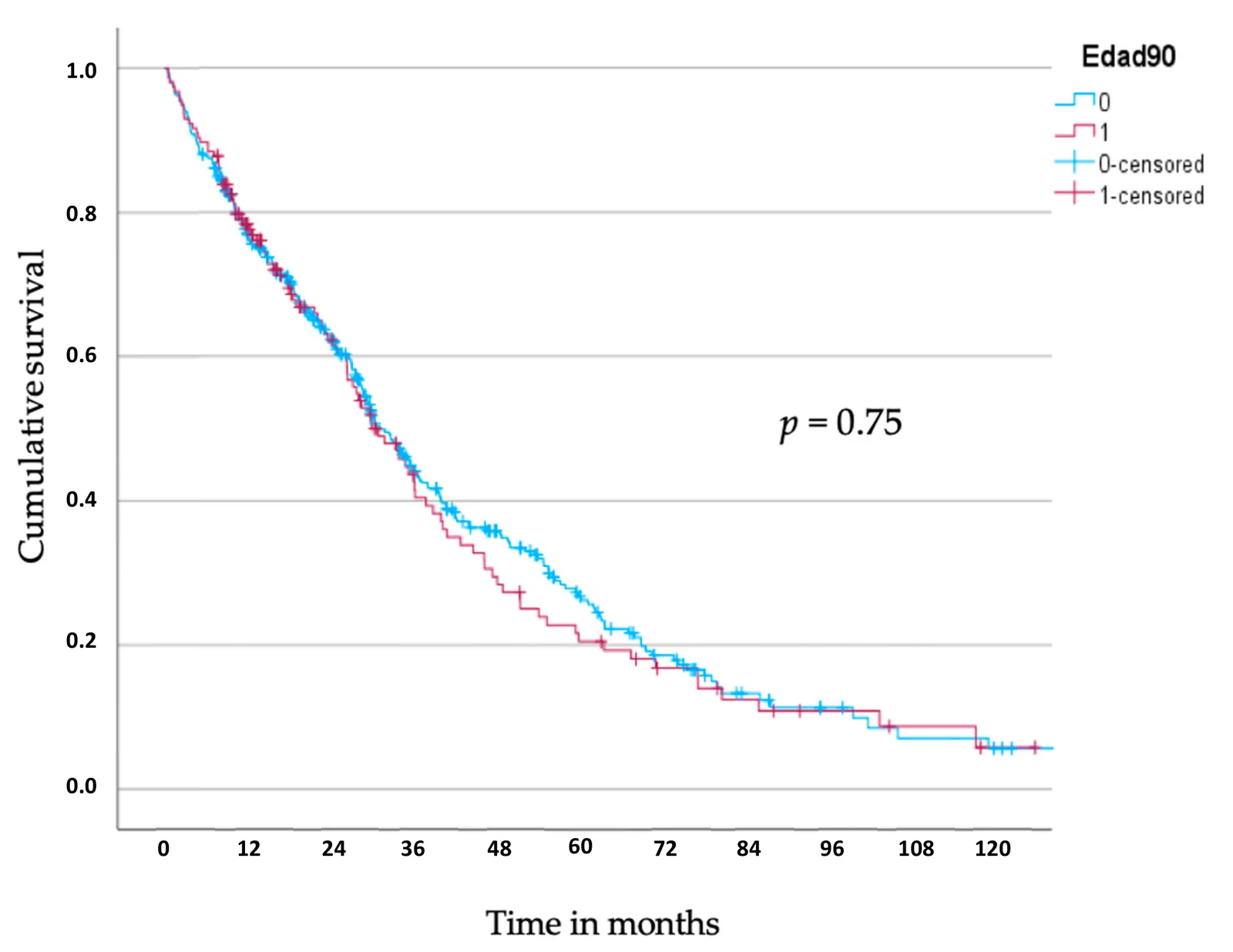
Relationship between survival and age.

Age was not significantly associated with survival, whether modelled per 5-year increment (HR 1.12; 95% CI 0.94–1.35) or comparing patients aged ≥90 versus 85–89 years (HR 1.05; 95% CI 0.83–1.33; log-rank *p* = 0.75) (Table 1; Figure 3).

Patients with new-onset heart failure (compared with chronic HF) had a better survival rate (HR: 0.77; 95% CI 0.59–0.99). The aetiologies of heart disease associated with lower survival were ischaemic (HR: 1.49; 95% CI 1.18–1.88) and infiltrative cardiomyopathy (HR: 1.72; 1.06–2.78) (Table 4).

A worse HF functional class (NYHA III–IV) and previous or subsequent hospital admissions for HF were associated with poorer survival. Thus, having NYHA functional class III–IV (HR: 1.47; 95% CI 1.18–1.83), previous HF admissions at the start of follow-up (HR: 1.37; 95% CI 1.09–1.71) and HF readmission within 1-year follow-up (HR: 3.05; 95% CI 2.10–4.44) were significantly associated with increased mortality (Table 4).

Similarly, higher NT-proBNP considered as a continuous log10-transformed variable was associated with shorter survival (HR: 2.49; 95% CI 1.96–3.17).

**Figure 4:**
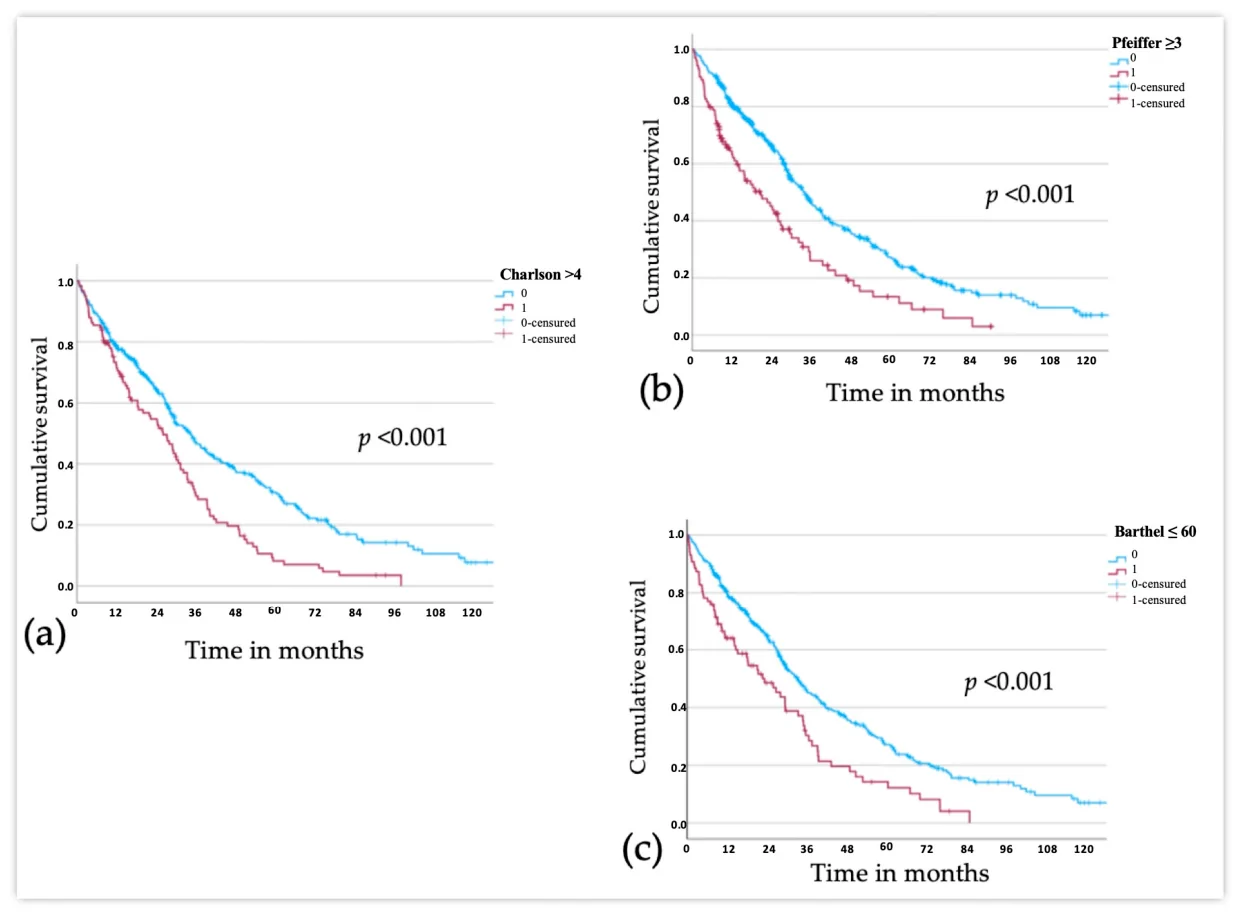
Kaplan–Meier survival curves according to Charlson index (>4) (**a**), Pfeiffer test (≥3) (**b**) and Barthel index (<60) (**c**).

Additionally, functional dependence (Barthel < 60 points) (HR: 1.70; 95% CI 1.30–2.23), cognitive impairment (Pfeiffer ≥ 3 errors) (HR: 1.81; 95% CI 1.40–2.34) and a higher comorbidity burden (Charlson index > 4 points) (HR: 1.66; 95% CI 1.31–2.10) were also associated with increased mortality (Table 2; Figure 4).

### 3.5. Multivariable Analysis of Predictors of Mortality

In multivariable models, overall comorbidity burden was represented by the Charlson index rather than by the individual comorbidities to avoid redundancy and potential collinearity. In the multivariable Cox regression analysis (Table 5), after adjusting for age, sex, and variables significantly associated with mortality in the univariate analysis, independent predictors of lower survival were cognitive impairment (higher Pfeiffer score) (HR: 1.07; 95% CI 1.00–1.14), comorbidity burden (higher Charlson index) (HR: 1.10; 95% CI 1.03–1.18), worse NYHA functional class (HR: 1.30; 95% CI 1.01–1.68) and high NT-proBNP (>2285 pg/mL) (HR: 2.03; 95% CI 1.57–2.62). Conversely, a better functional status (higher Barthel index) had an independent protective effect on survival (HR: 0.99; 95% CI 0.98–0.996).

When HF readmission was included in the multivariable model (318 patients with complete covariate data, 196 deaths), it was strongly associated with lower survival. In this model, only Charlson index (HR:1.101; 95% CI 1.011–1.189), and elevated NT-proBNP (HR:1.756; 95% CI 1.296–2.379) remained independently associated with mortality, while the Barthel index (HR: 1.00; 95% CI 0.99–1.00) showed a borderline association (Table 6).

## 4. Discussion

In this cohort of patients aged ≥85 years with heart failure (HF), long-term prognosis was poor, with an estimated survival of 50% at 2.5 years and 25% at 5 years. These outcomes appear less favourable than those reported in younger HF cohorts, in which survival ranges from approximately 65–69% at 3–5 years in primary-care populations (mean age 76–77 years) [21,22] and reaches 56.7% at 5 years in a meta-analysis of community-dwelling patients, declining progressively with age [23]. A large European registry of ambulatory patients with chronic HF reported a 5-year survival of approximately 68% in a substantially younger population (mean age ~67 years), and identified age as an independent predictor of mortality [24]. The less favourable prognosis in our cohort likely reflects its advanced age (mean 88 years) and the higher burden of multimorbidity and functional impairment typical of patients aged ≥85 years.

Data focusing specifically on long-term outcomes in very elderly HF patients remain scarce. A Swedish population-based cohort of patients aged ≥85 years with newly diagnosed heart failure [12] reported a 1-year mortality of 30.5%, which is comparable to the 23% observed in the present cohort. Consistently, Baldasseroni et al. reported a 1-year mortality of 22.7% in a cohort of very elderly patients (mean age 89 years) managed in a multidisciplinary heart failure unit after acute decompensation [13].

Consistent with the phenotype of very elderly heart failure, HF with preserved ejection fraction predominated. This pattern is also observed in internal-medicine registries, in which HFpEF represents a substantial proportion of hospitalised heart failure cases and is typically associated with older age and higher comorbidity burden [25,26].

Patients exhibited a high burden of comorbidity. Hypertension was the most prevalent condition, affecting nearly all patients (95.9%); this exceeds the rates reported in most series (50–90%) [10,12,27] and likely reflects the advanced age of the cohort. Atrial fibrillation and chronic kidney disease were also highly prevalent, affecting approximately two thirds and more than half of patients, respectively, with rates higher than those reported in most studies [12,25,28], but comparable to those observed in the Spanish RICA registry [27].

Regarding factors related to **survival**, no relationship between **age** and survival was observed. Although such an association has been consistently reported in cohorts including younger patients [21,22,23,24,29], chronological age appears to lose prognostic relevance in very elderly populations, as also described in octogenarians [14,30]. In cohorts restricted to very elderly patients, age may lose prognostic significance relative to markers of biological age, such as multimorbidity, functional dependence, and cognitive impairment. Likewise, Baldasseroni et al. identified residual congestion and social isolation (living alone), rather than age itself, as independent predictors of 1-year mortality in a cohort of very elderly patients with HF [13]. Similarly, in a large multicentre cohort of patients hospitalised for acute HF, age was not associated with mortality among patients aged ≥85 years, whereas factors such as delirium and overall clinical assessment were stronger predictors of short-term outcomes [14]. Nonetheless, this may partly be due to the narrow age range of the cohort, in which all patients were aged ≥85 years (median 88 years, most between 86 and 90), which limits the statistical power to detect small age-related differences in survival.

No association between **sex** and survival was observed in our cohort. There is some controversy in the literature in this regard, since some studies support, as in our series, that there is no association [24], but others suggest that male sex is a risk factor for worse survival [27,31].

It is noteworthy that **chronic kidney disease** was not associated with survival, whereas most publications show that renal failure is an independent predictor of mortality in patients with heart failure [7,21,29]. However, advanced chronic kidney disease (eGFR < 30 mL/min/1.73 m^2^) was associated with significantly worse survival. This threshold effect is consistent with prior observations that severe renal impairment concentrates risk in heart failure and may reflect a cardio-renal frailty phenotype [32]. **Diabetes mellitus** was associated with worse survival. This is consistent with previous observational studies [10] and with meta-analyses linking diabetes to a higher risk of all-cause death and cardiovascular death [7,33,34].

**Dementia** and worse cognitive status (Pfeiffer test) were also associated with decreased survival as previously described [25]. Cognitive impairment plausibly contributes to worse outcomes through reduced self-care capacity and poorer adherence, and has been linked to impaired heart failure self-management [7,35,36]. **Ischaemic heart disease** was significantly associated with increased mortality due to heart failure, thus supporting what has been widely described in the literature [21,22,29]. The coexistence of **neoplasia** worsened the prognosis, aligning with evidence that cancer coexisting with HF is associated with poor outcomes and higher mortality [37].

Beyond the impact of individual comorbidities on survival, a higher overall **comorbidity burden**, assessed by the Charlson index, was independently associated with poorer survival. This association is well established [7,38].

The limited use of contemporary disease-modifying therapies in this cohort should be interpreted in the context of the long recruitment period and the evolving therapeutic landscape of heart failure. SGLT2 inhibitors were incorporated into guideline recommendations for heart failure with reduced ejection fraction only in 2021 [15] and extended across the full spectrum of left ventricular ejection fraction in the 2023 focused update [39], at the very end of our recruitment period. In addition, only 13.6% of patients had reduced ejection fraction, limiting the proportion of patients for whom ARNI was guideline-recommended on the basis of heart failure phenotype during the study period. Evidence regarding the use of contemporary disease-modifying therapies in patients of very advanced age remains comparatively limited, and suboptimal uptake has also been reported in cohorts of patients aged ≥80 years [40]. Further studies specifically addressing the implementation, tolerability, and clinical impact of these therapies in patients of very advanced age are warranted.

Markers of HF severity such as higher **NT-proBNP levels** and worse **NYHA functional class** (III–IV) were associated with worse prognosis as reported in previous studies [12,41,42,43]. In the baseline multivariable model (Table 5), both remained independently associated with mortality.

In addition, **functional status** (Barthel index) and **cognitive status** (Pfeiffer test) were independent predictors of survival in the baseline multivariable model (Table 5). These findings support a framework in which, among patients aged ≥85 years, prognosis is driven less by chronological age than by the interaction between heart failure severity, multimorbidity, functional dependence, and cognitive impairment. This reinforces the need for a multidimensional geriatric approach to care.

Finally, the prognostic impact of **HF readmissions** was strong when included in the multivariable model (Table 6): readmission within the first year was the strongest predictor of mortality, while the associations of baseline functional class, cognitive status, and functional dependence were attenuated. In contrast, the Charlson index and high NT-proBNP remained independently associated with mortality, representing the most consistent prognostic markers across the two models. The strong influence of readmissions on survival has also been highlighted by different authors [44]. The ESC-EORP-HFA Heart Failure Long-Term Registry, carried out in 211 centres and including 12,440 patients, corroborates the severity of HF once admission is required. In that registry, almost one third of patients died in the first year after admission and almost half were readmitted for heart failure [45]. These findings highlight the importance of early identification of high-risk patients and the potential role of structured follow-up programmes in preventing adverse outcomes. In this cohort, the highest risk of death was identified by markers of biological age and disease severity, not by chronological age. These markers included comorbidity burden, functional and cognitive status, natriuretic peptide levels and early HF readmission. A multidimensional assessment based on these domains may therefore be more informative than age alone when estimating prognosis and tailoring the intensity of follow-up in this population.

This study has several limitations inherent to its retrospective design, including potential information bias and missing data for some variables. Because the multivariable models were fitted using complete-case analysis, patients with missing covariate data—most notably 1-year HF readmission, missing in 38.1% of the sample—were excluded from the respective models, reducing the effective sample size and potentially introducing selection bias. In addition, the single-centre nature of the study may limit the generalisability of the findings. Moreover, patients were referred on the basis of clinical judgement and had to be able to attend the outpatient unit. Consequently, the study population tended to include patients with more advanced heart failure but preserved functional status, which may limit the representativeness of the cohort. Data on socio-economic status and caregiver support were also unavailable. Unmeasured confounding by these factors, potentially relevant in this age group, cannot be excluded. Information on previous coronary revascularisation, valve interventions or prostheses, and cardiac implantable electronic devices was not systematically collected and could therefore not be included in the analyses. As these interventions may influence outcomes, their omission represents a further potential source of residual confounding, and their prognostic impact in patients aged ≥85 years with heart failure warrants further investigation. The absence of formal frailty assessment may have limited the evaluation of biological age and vulnerability. Finally, the prognostic effects of the main covariates (comorbidity burden, functional class, functional dependence and NT-proBNP) were not constant over time, attenuating during a prolonged follow-up; the corresponding hazard ratios should therefore be interpreted as average effects over the study period rather than as time-invariant estimates.

However, this study includes a relatively large sample of very elderly patients with HF managed within a Comprehensive Heart Failure Management Unit (UMIPIC) integrated into a national network and following a standardised care model [10]. This setting provides valuable insight into a frequently underrepresented and clinically complex geriatric population, particularly regarding survival and its associated factors. Moreover, the structured and multidisciplinary nature of this care model may enhance the applicability and generalisability of the findings to comparable HF units, and could help inform clinical decision-making regarding the management and follow-up of very elderly patients with heart failure.

## 5. Conclusions

In this cohort of patients aged ≥85 years with heart failure, long-term survival was poor, with approximately half alive at 2.5 years and only one quarter at 5 years. Prognosis appeared to be more closely associated with clinical complexity than with chronological age. Comorbidity burden assessed using the Charlson Comorbidity Index, functional and cognitive status assessed using the Barthel Index and Pfeiffer test, NYHA functional class, and NT-proBNP were independent predictors of mortality. Early HF readmission emerged as the strongest predictor when included in the model. These findings suggest that a comprehensive multidimensional assessment incorporating these domains may be useful in the management and prognostic evaluation of this population. The prognostic contribution of contemporary disease-modifying therapies and of cardiovascular interventions and devices could not be assessed in this cohort and warrants further investigation.

## Figures and Tables

**Table 1:** Demographic characteristics and comorbidities of survivors versus deceased patients.

	Total *N* = 514	Alive *N* = 174 (33.9%)	Dead *N* = 340 (66.1%)	*p*-Value	HR * (95% CI)
Demographic data					
Age (years)				0.207	1.02 (0.99–1.06)
Median [IQR *]	88 [86–90]	88 [86–90]	88 [86–90]		
Mean (SD *)	88.4 (3.0)	88.52 (3.0)	88.4 (3.0)		
Sex:				0.757	0.97 (0.78–1.20)
Male	237 (46.1%)	77 (44.3%)	160 (47.1%)		
Female	277 (53.9%)	97 (55.7%)	180 (52.9%)		
Lives alone	85 (17.7%)	30 (18.3%)	55 (17.4%)	0.995	1.00 (0.75–1.34)
	*N* = 480	*N* = 164	*N* = 316		
Lives with spouse	126 (27.8%)				
	*N* = 454				
Institutionalised	11 (2.3%)	2 (1.2%)	9 (2.8%)	0.493	1.26 (0.65–2.45)
	*N* = 487	*N* = 167	*N* = 321		
Comorbidities					
Hypertension	493 (95.9%)	165 (94.8%)	328 (96.5%)	0.957	1.02 (0.57–1.81)
**Diabetes mellitus**	250 (48.6%)	79 (45.4%)	171 (50.3%)	**0.038**	**1.26 (1.01–1.56)**
Dyslipidaemia	294 (57.2%)	95 (54.6%)	199 (58.5%)	0.530	1.07 (0.86–1.33)
Obesity	173 (34.6%)	65 (37.8%)	108 (32.9%)	0.109	0.83 (0.66–1.04)
CKD *	294 (57.3%)	96 (55.5%)	198 (58.2%)	0.164	1.17 (0.94–1.45)
	*N* = 513	*N* = 173	*N* = 340		
Advanced CKD * eGFR * < 30 mL/min/1.73 m^2^	95 (19.3%)	22 (12.9%)	73 (22.7%)	**0.008**	**1.99 (1.19–3.35)**
	*N* = 492	*N* = 171	*N* = 321		
Stroke	57 (11.1)	22 (12.7%)	35 (10.3%)	0.052	1.42 (0.997–2.02)
	*N* = 513	*N* = 173	*N* = 340		
**Acute myocardial infarction**	105 (20.5)	28 (16.3%)	77 (22.6%)	**0.017**	**1.36 (1.06–1.76)**
	*N* = 512	*N* = 172	*N* = 341		
Peripheral arterial disease	39 (7.6)	13 (7.5%)	26 (7.6%)	0.367	1.20 (0.81–1.80)
	*N* = 513	*N* = 173	*N* = 342		
Atrial fibrillation	353 (68.7)	121 (69.5%)	232 (68.2%)	0.925	1.01 (0.81–1.27)
COPD *	82 (16.0%)	21 (12.51%)	61 (18%)	0.103	1.26 (0.95–1.66)
	*N* = 513	*N* = 166	*N* = 339		
**Dementia**	83 (16.2%)	15 (8.6%)	68 (20.1%)	**<0.001**	**1.77 (1.36–2.31)**
	*N* = 513	*N* = 174	*N* = 338		
**Cancer**	38 (7.4%)	5 (2.9%)	33 (9.7%)	**0.007**	**1.65 (1.15–2.36)**
	*N* = 513	*N* = 172	*N* = 340		
Anaemia	284 (55.4%)	99 (56.9%)	185 (54.6%)	0.173	1.16 (0.94–1.44)
	*N* = 513	*N* = 174	*N* = 339		
Liver disease	40 (7.8%)	10 (5.8%)	30 (8.8%)	0.212	1.27 (0.87–1.85)
	*N* = 512	*N* = 172	*N* = 341		

* HR: hazard ratio; IQR: Interquartile range; SD: standard deviation; CKD: chronic kidney disease; eGFR: estimated glomerular filtration rate; COPD: chronic obstructive pulmonary disease; bold format: variables that were statistically significant, together with their corresponding *p*-values and hazard ratios.

**Table 2:** Functional status, cognitive status, and global comorbidity assessed by standardised scales.

	Total *N* = 514	Alive *N* = 174 (33.9%)	Dead *N* = 340 (66.1%)	*p*-Value	HR (95% CI)
**Barthel Index**					
Median [IQR *] Mean (SD *)	85 [65–100] 79.04 (21.4)	90 [75–100] 83.57 (19.7)	80 [65–95] 76.7 (21.9)	**<0.001**	**0.99 (0.98–0.99)**
Barthel < 60, *n* (%)	87 (17.3%) *N* = 504	19 (11.1%) *N* = 171	68 (20.4%) *N* = 333	**<0.001**	**1.70 (1.30–2.23)**
**Pfeiffer test**					
Median [IQR *] Mean (SD *)	0 [0–2] 1.16 (2)	0 [0–1] 0.98 (1.8)	0 [0–2] 1.26 (2.05)	**<0.001**	**1.12 (1.07–1.18)**
Pfeiffer ≥ 3 errors, *n* (%)	104 (21.5%) *N* = 484	28 (17.4%) *N* = 161	76 (23.5%) *N* = 323	**<0.001**	**1.81 (1.40–2.34)**
**Charlson Index**					
Median [IQR *] Mean (SD *)	3 [2,3,4] 3.39 (1.8)	3 [2,3,4] 2.87 (1.46)	3 [2,3,4,5] 3.66 (1.9)	**<0.001**	**1.17 (1.11–1.23)**
Charlson > 4, *n* (%)	124 (24.1%)	25 (14.4%)	99 (29.1%)	**<0.001**	**1.66 (1.31–2.10)**

* IQR: Interquartile range; SD: standard deviation; bold format: variables that were statistically significant, together with their corresponding *p*-values and hazard ratios.

**Table 3:** Baseline medical therapy.

	*N* = 514
Beta-blockers (*N* = 505)	314 (62.2%)
Angiotensin-Converting Enzyme inhibitors—ACEi (*N* = 505)	56 (11.1%)
Angiotensin II receptor blockers—ARBs (*N* = 504)	126 (25%)
Angiotensin receptor–neprilysin inhibitors—ARNI (*N* = 505)	52 (10.3%)
Antialdosteronics (*N* = 505)	213 (42.2%)
Sodium–Glucose Cotransporter 2 inhibitors—SGLT2i (*N* = 505)	114 (22.6%)
Loop diuretics (*N* = 505)	453 (89.7%)
Thiazide diuretics (*N* = 505)	45 (8.9%)
Anticoagulants (*N* = 504)	302 (59.9%)
Antiplatelets agents (*N* = 504)	154 (30.55%)
Statins (*N* = 503)	266 (52.9%)

**Table 4:** Characteristics and evolution of heart failure in survivors versus deceased patients.

		Total *N* = 514	Alive *N* = 174 (33.9%)	Dead *N* = 340 (66.1%)	*p*-Value	HR * (95% CI)
HF * characteristics						
LVEF *					0.678	1.00 (0.99–1.01)
Median [IQR *]		60 [47.25–66]	58 [45–67]	60 [48–66]		
Mean (SD *)		56.24 (14)	55.78 (14)	56.49 (14)		
LVEF *	Reduced	70 (13.6%)	28 (16.2%)	42 (12.7%)	0.071	1.35 (0.98–1.87)
	Mildly reduced	53 (10.3%)	17 (9.8%)	36 (10.9%)	0.72	1.12 (0.61–2.06)
	Preserved	380 (73.9%)	128 (74%)	252 (76.1%)	0.60	1.21 (0.73–1.71)
	Not available	11 (2.1%)	—	—	—	—
New-onset HF *		130 (25.3%)	59 (33.9%)	71 (20.9%)	**0.043**	**0.77 (0.59–0.99)**
NYHA * III–IV		182 (36.8%)	40 (24.0)	142 (43.3)	**<0.001**	**1.47 (1.18–1.83)**
		*N* = 495	*N* = 167	*N* = 328		
NT-proBNP * (log_10_) pg/mL Median [IQR *]		3.46 [3.10–3.79]	3.25 [2.96–3.57]	3.57 [3.24–3.90]	**<0.001**	**2.49 (1.96–3.17)**
Heart disease aetiology						
Hypertensive		393 (78.6%)	134(77.9%)	259 (79.0%)	0.224	0.85 (0.65–1.11)
		*N* = 500	*N* = 172	*N* = 328		
**Ischaemic**		147 (29.3%)	37 (21.5%)	110 (33.4%)	**<0.001**	**1.49 (1.18–1.88)**
		*N* = 501	*N* = 172	*N* = 329		
Valvular		205 (40.8%)	75 (43.4%)	130 (39.5%)	0.061	1.24 (0.99–1.54)
		*N* = 502	*N* = 173	*N* = 329		
**Infiltrative**		28 (5.6%)	10 (5.8%)	18 (5.5%)	**0.028**	**1.72 (1.06–2.78)**
		*N* = 500	*N* = 172	*N* = 327		
Clinical course						
Prior HF * admission		323 (63.3%)	103 (59.9%)	220 (65.1%)	**0.006**	**1.37 (1.09–1.71)**
		*N* = 510	*N* = 172	*N* = 338		
HF * admission in the year prior to follow-up		269 (53.1%)	88 (50.9%)	181 (54.2%)	**0.016**	**1.30 (1.05–1.62)**
		*N* = 507	*N* = 173	*N* = 334		
HF * readmissions		43 (11.4%)	9 (6.2%)	34 (14.7%)	**<0.001**	**3.05 (2.10–4.44)**
		*N* = 377	*N* = 145	*N* = 231		

* HR: hazard ratio; HF: heart failure; LVEF: left ventricular ejection fraction; IQR: interquartile range; SD: standard deviation; NYHA: New York Heart Association; NT-proBNP: N-terminal pro-B-type natriuretic peptide; bold format: variables that were statistically significant, together with their corresponding *p*-values and hazard ratios.

**Table 5:** Multivariable analysis of factors related to survival.

	B	*p*-Value	HR (95% CI)
Age	0.000	0.988	1.00 (0.96–1.04)
Sex	−0.082	0.529	0.92 (0.72–1.19)
**Pfeiffer test**	0.066	**0.048**	**1.07 (1.00–1.14)**
**Barthel Index**	−0.010	**0.001**	**0.99 (0.98–0.996)**
**Charlson Index**	0.096	**0.004**	**1.10 (1.03–1.18)**
Ischaemic aetiology	0.108	0.450	1.11 (0.84–1.69)
New-onset HF *	0.096	0.557	1.10 (0.80–1.51)
NYHA * III–IV	0.261	**0.044**	**1.30 (1.01–1.68)**
High NT-proBNP * levels (>2285 pg/mL)	0.706	**<0.001**	**2.03 (1.57–2.62)**

Model based on 425 patients with complete covariate data, including 277 deaths. * HF: heart failure. NYHA: New York Heart Association. NT-proBNP: N-terminal pro-B-type natriuretic peptide; bold format: variables that were statistically significant, together with their corresponding *p*-values and hazard ratios.

**Table 6:** Multivariable analysis of factors related to survival including readmissions.

	B	*p*-Value	HR (95% CI)
Age	0.022	0.429	1.02 (0.97–1.08)
Sex	0.020	0.900	0.98 (0.72–1.33)
Pfeiffer test	0.070	0.111	1.07 (0.98–1.17)
Barthel Index	0.007	0.071	0.99 (0.99–1.00)
**Charlson Index**	0.092	**0.027**	**1.10 (1.01–1.19)**
Ischaemic aetiology	0.226	0.183	1.25 (0.90–1.75)
New-onset HF *	0.186	0.343	1.20 (0.82–1.77)
NYHA * III–IV	0.121	0.445	1.13 (0.83–1.54)
High NT-proBNP * levels **(>2285 pg/mL)**	0.563	**<0.001**	**1.76 (1.30–2.38)**
HF * readmissions within the 1st year	0.984	**<0.001**	**2.68 (1.72–4.16)**

Model based on 318 patients with complete covariate data, including 196 deaths. * HF: heart failure. NYHA: New York Heart Association. NT-proBNP: N-terminal pro-B-type natriuretic peptide; bold format: variables that were statistically significant, together with their corresponding *p*-values and hazard ratios.

## Data Availability

The data presented in this study are not publicly available due to privacy and ethical restrictions but are available from the corresponding author upon reasonable request.

## Abbreviations

The following abbreviations are used in this manuscript:

HF	Heart Failure
UMIPIC	Comprehensive Management Units for Patients with Heart Failure (UMIPIC programme)
CKD	Chronic Kidney Disease
LVEF	Left Ventricular Ejection Fraction
NYHA	New York Heart Association
NT-proBNP	N-terminal pro-B-type natriuretic peptide
SD	Standard Deviation
IQR	Interquartile Range
HR	Hazard Ratio
SPSS	Statistical Package for the Social Sciences
DM	Diabetes Mellitus
AMI	Acute Myocardial Infarction
PAD	Peripheral Arterial Disease
AF	Atrial Fibrillation
COPD	Chronic Obstructive Pulmonary Disease
eGFR	Estimated Glomerular Filtration Rate
CI	Confidence Interval
